# The potential of DeepSeek for AI-aided diagnosis of antibody-positive autoimmune encephalitis: a single-center, retrospective, observational study

**DOI:** 10.3389/frai.2025.1638904

**Published:** 2025-10-06

**Authors:** Huanyu Meng, Yihua Tang, Yuanqi Qi, Qinming Zhou, Lu He, Sheng Chen

**Affiliations:** ^1^Department of Neurology, Ruijin Hospital, Shanghai Jiao Tong University School of Medicine, Shanghai, China; ^2^Department of Neurology, Xinrui Hospital, Wuxi, China; ^3^Clinical Center for Rare Diseases, Ruijin Hospital, Shanghai Jiao Tong University School of Medicine, Shanghai, China; ^4^Department of Neurology, The First People’s Hospital of Fuyang, Hangzhou, China; ^5^Co-Innovation Center of Neuroregeneration, Nantong University, Nantong, China

**Keywords:** autoimmune encephalitis, DeepSeek, artificial intelligence, MOG, GABAbR

## Abstract

**Background:**

Autoimmune encephalitis (AIE) is challenging to diagnose, especially in primary hospitals in China with limited medical resources. DeepSeek, a newly developed AI, shows potential as a cost-effective tool for improving diagnostic efficiency. However, no studies have evaluated the diagnostic accuracy of DeepSeek for AIE.

**Methods:**

This retrospective study included 100 patients with anti-neuronal antibody-positive AIE treated at Ruijin Hospital, Shanghai Jiao Tong University School of Medicine. After removing personally identifiable information, antibody results, and history of immunotherapy from patients’ medical histories, the following information was sequentially input into DeepSeek: sex, age, chief complaint, medical history, EEG findings, head MRI description, and cerebrospinal fluid (CSF) results. The positive rates of AIE diagnoses predicted by DeepSeek were then categorized as most likely diagnosis, differential diagnosis, and total diagnosis.

**Results:**

Using DeepSeek, the probabilities of AIE appearing as the most likely diagnosis and total diagnosis accuracy were 49 and 65%. When patient data were input stepwise, both the total diagnosis accuracy and the most likely diagnosis accuracy did not significantly increase. AIE patients with anti-MOG and anti-GABAbR positivity had predicted total diagnostic positivity rates of 88 and 100%, respectively. Patients presenting with headache and epilepsy were more likely to be diagnosed with AIE (96 and 100%).

**Conclusion:**

DeepSeek shows limited positive diagnostic accuracy for predicting the diagnosis of AIE. The application of this new AI technology could be used to promote early screening for AIE in primary hospitals in China, improve medical education, and lead to research advances in AIE.

## Introduction

Autoimmune encephalitis (AIE) is a central nervous system (CNS) autoimmune disease characterized by subacute onset and diverse symptoms including psychiatric and behavioral abnormalities, cognitive impairments, seizures, and ataxia (Graus et al., 2016). These symptoms significantly impact patients’ quality of life and lead to a severe disease burden. Despite the introduction of diagnostic guidelines for AIE, such as those by Graus et al. (2016) and Dalmau and Graus (2023) diagnostic criteria specifically addressing the diagnosis of antibody-negative AIEs (Dalmau and Graus, 2023), diagnosis is often delayed due to symptom variability, the complexity of differential diagnosis, and the time required for antibody testing. Previous studies have shown that AIE prognosis is closely related to receiving early, effective treatment, which necessitates early, efficient diagnosis (Dong and Yi, 2023). Therefore, methods to assist diagnosis of AIE are critical for improving prognosis.

Rapid advancements in artificial intelligence (AI) technology have led to their rapidly growing role in disease diagnosis (Kumar et al., 2023). Tools like OpenAI’s ChatGPT (Liu et al., 2024), PathChat (Lu et al., 2024), and Microsoft’s Copilot (Daza et al., 2024) have far surpassed their original scope as language models and now significantly contribute to medical diagnostics and decision-making across (Dave et al., 2023). A study using ChatGPT to diagnose CNS disorders based on 100 consecutive “Case of the Week” reports published in the *American Journal of Neuroradiology* between October 2021 and September 2023 found it could provide a diagnosis in 50% of cases (Horiuchi et al., 2024). DeepSeek is a newly developed, large-scale AI platform based on large language model (LLM) technology (Denecke et al., 2024). While many other LLM-based tools (e.g., ChatGPT) have demonstrated diagnostic capabilities in prior studies (Mykhalko et al., 2023), there has been no published research validating the performance of DeepSeek in real-world healthcare applications. Since early 2025, there has been increasing research interest in comparing the abilities of DeepSeek and ChatGPT in disease diagnosis (Kayaalp et al., 2025; Peng et al., 2025). However, the diagnostic accuracy of an AI tool for AIE is yet to be established.

To address this research gap, we performed a stratified analysis using DeepSeek inputting the sex, age, medical history, MRI findings, EEG descriptions, cerebrospinal fluid (CSF) nucleated cell count, and CSF protein levels of patients with antibody-positive AIE. The goal of this study was to determine the accuracy and potential applications of DeepSeek for diagnosing AIE, providing a theoretical foundation for future research on AI-assisted diagnosis of this condition.

## Methods

### Participants

This single-center prospective study was approved by the Ethics Committee of Ruijin Hospital, Shanghai Jiao Tong University School of Medicine [approval number: 2021(374)]. All patients provided written informed consent.

**Figure 1:**
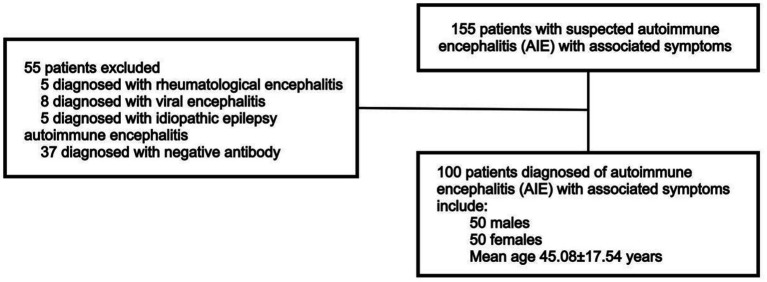
Flowchart of patient inclusion and exclusion criteria.

The analysis included 100 selected antibody-positive patients with AIE treated at Ruijin Hospital, affiliated with Shanghai Jiao Tong University School of Medicine, between March 15, 2022, and February 15, 2025. These 100 patients were retrospectively selected based on the availability of complete clinical data and confirmed antibody positivity. These criteria were necessary to minimize potential bias caused by missing diagnostic data. The diagnostic criteria for AIE follow those established by Graus et al. (2016). Accordingly, patients with symptoms associated with COVID-19 recovery or brain fog caused by chemotherapy or polypharmacy were excluded. Patients’ medical histories were screened to confirm positive results for the following antibodies in serum and CSF, which are indicative of AIE: NMDAR, CASPR2, LGI1, AMPAR1, AMPAR2, GABAbR, GlyRa1, DPPX, IgLON5, mGluR1, mGluR5, D2R, NRXN3A, KLHL11, GluK2, AK5, VGCC, MOG, GFAP, and onconeural antibodies (Hu, Yo, Ri, Ma2, Ma1, CV2/CRMP5, amphiphysin, Tr/DNER, SOX1, Titin, Zic4, Recoverin, PKC γ, and GAD65). All antibody results were verified using a dual-confirmation approach. Antibodies detected by either cell-based assay (CBA) or immunoblot (BLOT) were further confirmed by tissue-based assay (TBA) whenever possible, ensuring higher diagnostic confidence. Only patients double-positive for antibodies in serum and CSF, with titers ≥1:100 in both compartments, as measured by CBA and who exhibited clinical symptoms were included in the analysis (Figure 1).

### Study design

This study was designed according to the Standards for Reporting Diagnostic Accuracy Studies (STARD) statement. Data for each patient, including sex, age, chief complaint, medical history, EEG findings, head MRI description, CSF nucleated cell count, and CSF protein levels, were obtained from medical records. These data were input to DeepSeek in a stratified manner, and we then extracted the differential and most likely diagnoses generated. To simulate the clinical scenario of a patient’s initial presentation, we selected the most readily available clinical symptoms and ancillary test results in routine practice to make diagnostic inferences. Specifically, we input the basic data (sex, age, chief complaint, medical history, vaccination or infection history), MRI description, EEG description, and CSF analysis results for the same patient in four separate steps to assess whether DeepSeek’s diagnostic accuracy varied with different information. This also allowed us to explore patterns that allow for accurate diagnoses with minimal input.

### Input and output procedure for DeepSeek

**Figure 2:**
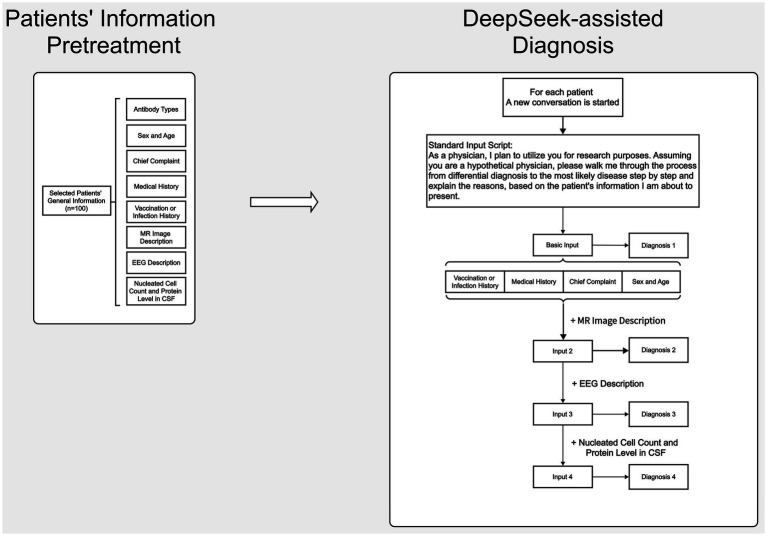
Flowchart of clinical data collection for antibody-positive AIE patients and step-by-step input of clinical data to the DeepSeek interface.

**Figure 3:**
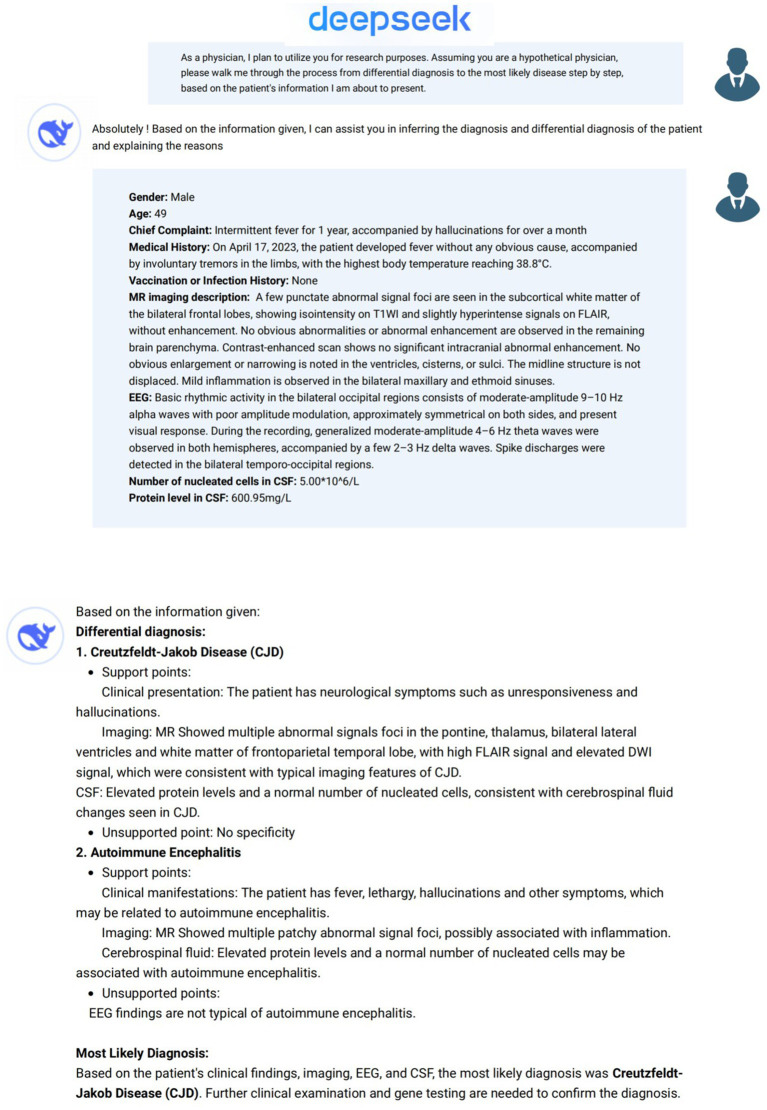
Patient information input and operation method using the DeepSeek interface.

Analysis of patient data using DeepSeek was performed between February 15, 2025 and March 5, 2025 (Figures 2, 3). To provide background for our request, the following description was first entered into DeepSeek-V3 (2024-12-26 Version, https://chat.deepseek.com/): *As a physician, I plan to utilize you for research purposes. Assuming you are a hypothetical physician, please walk me through the process from differential diagnosis to the most likely disease step by step, based on the patient information I am about to present* (Horiuchi et al., 2024). We then input the general information, MRI description, EEG description, and CSF analysis results (nucleated cell count and CSF protein levels) for each patient in four separate steps. In line with institutional ethical standards and standard research protocols, we removed any personally identifiable information from each patient’s data and any details regarding antibody testing or immunosuppressive treatment that might strongly suggest AIE. As the primary aim of our study was to simulate a real-world clinical scenario, particularly at the early diagnostic stage in emergency or outpatient settings where clinicians often make decisions with incomplete data. Thus, we deliberately excluded diagnostic clues such as antibody test results, antibody titers, CSF next-generation sequencing (NGS), and treatment response from the input data. This process ensured that the input data described only the patient’s clinical symptoms and paraclinical data (e.g., MRI, EEG, and CSF findings). The formal EEG and MRI descriptive reports issued by senior clinical neurophysiology and radiology experts, not the final EEG or MRI conclusions, were input. To ensure consistency and quality across cases, all input data were reviewed in two rounds by senior EEG and radiology specialists. The revised medical history input data were reviewed and approved by a senior neurologist with 20 years of clinical experience (CS). The differential diagnoses and most likely diagnosis provided by DeepSeek as answers to each step were extracted. A new DeepSeek session was initiated for each case to avoid the influence of prior answers on the output.

### Output evaluation

The DeepSeek output included several differential diagnoses and the most likely diagnoses. Two neurologists (HM and QZ) assessed whether the differential diagnoses or the most likely diagnosis generated by DeepSeek included AIE. To consider DeepSeek’s assessment successful, the generated text needed to explicitly contain the term “autoimmune encephalitis.” In case of any disagreements relating to the evaluation, the final decision was made by a senior neurologist with 20 years of experience (CS). The total diagnosis accuracy rate or total positive rate was defined as the ratio of the number of patients for whom “autoimmune encephalitis” appeared in either the most likely diagnosis or differential diagnosis to the total number of patients included in the analysis.

### Statistical analysis

Statistical analyses were performed using Graphpad prism software and SPSS 20.0 (IBM Corp.). The chi-square test and Fisher’s exact test (when *n* < 10) were conducted to compare the positive rates of the most likely, differential, and total diagnoses for AIE. Statistical significance for this exploratory analysis was set at *p* < 0.05.

## Results

### General patient information

Of the 100 patients with antibody-positive AIE included in this study, 50% were male, and the average age was 45.08 ± 17.54 years. The most common clinical symptoms were mental behavior disorder (42%), epilepsy (38%), and cognitive disorder (25%). The most common antibody was anti-NMDAR (22%), followed by anti-LGI1 (19%). The antibody types were confirmed by a combination of serum, CSF, and TBA analyses. Four patients (4%) had a history of infection or vaccination prior to symptom onset. In terms of neuroimaging findings, 72 of 81 (89%) patients exhibited some abnormal MRI signal, and 61 of 64 patients exhibited some EEG abnormalities; while these abnormalities may relate to AIE, some could be due to other causes. Additionally, 29 of 66 (44%) patients had CSF with abnormal nucleated cell counts (≥5 cells per mm^3^), and 27 of 66 (41%) had significantly elevated CSF protein levels (≥450 mg/L; Table 1).

**Table 1:** Characteristics of patients diagnosed with antibody-positive AIE.

Characteristic	Category	Patients diagnosed with antibody-positive AIE (*N* = 100)
Sex	Male	50
	Female	50
Age		45.08 ± 17.54
Clinical manifestations	Mental behavior disorder	42
	Epilepsy	38
	Cognitive disorder	25
	Dystonia	15
	Cerebellar ataxia	14
	Limb weakness	13
	Sleep disorder	12
	Speech disorder	6
	Visual impairment	5
	Autonomic dysfunction	6
	Consciousness disorder	2
	Fever	27
	Headache	18
Preceding infection or vaccination history^e^	Yes	4
	No	96
Antibody type	Anti-NMDAR	22
	Anti-LGI1	19
	Anti-MOG	15
	Anti-CASPR2	10
	Anti-GFAP	8
	Anti-GABAbR	7
	Anti-GAD65	6
	Anti-DPPX	5
	Anti-IgLON5	4
	Anti-Tr/DNER	2
	Anti-AK5	2
	Anti-mGluR1	2
	Anti-SEZ6L2	2
	Anti-SOX1	1
	Anti-GQ1b	1
	Anti-D2R	1
	Anti-mGluR5	1
MRI result	Positive^a^	72
	Negative^b^	9
EEG result	Positive^c^	61
	Negative^d^	3
Nucleated cell counts in CSF	≥5 cells per mm^3^	29
	0 < 5 < cells per mm^3^	37
Protein level in CSF	≥450 mg/L	27
	150–450 mg/L	39

a A positive MRI result is defined as follows: radiological abnormalities consistent with AIE (e.g., T2/FLAIR hyperintensities in limbic regions).

b A negative MRI result is defined as follows: “The MRI scan reveals no obvious abnormal signals within the brain in T1, T2 FLAIR, DWI, or T1 with contrast. The enhanced scan shows no distinct abnormal enhancement foci within the brain. No significant dilation or narrowing of the ventricles, cisterns, or sulci is noted. The midline structure is not displaced.”

c A positive EEG result is defined as follows: findings suggestive of encephalopathy or epileptic activity relevant to AIE.

d A negative EEG result is defined as follows: “The background rhythm is observed with adequate modulation and amplitude regulation, symmetrical bilaterally, and the visual response is negative.”

^e^A preceding history of infection or vaccination is defined as follows: “The patient self-reports a history of vaccination within the past 3 months or a history of infection within the last 1 month, with well-documented medical records confirming the history.”

The total number of some statistical indicators exceeding 100 is due to the fact that the same patient may have multiple clinical symptoms or multiple antibody types. The number of patients with MRI, EEG, and CSF results being less than 100 is because some patients have missing clinical data.

### Predictive diagnoses by DeepSeek after inputting patients’ clinical information

Each patient’s clinical information was input into DeepSeek in a stratified manner, as described above. We then recorded the incidence of AIE as the most likely diagnosis and differential diagnosis. Since this is a retrospective, observational, real-world study, data were missing for some patients. As a result, the accuracy rate may have a denominator that does not reach 100.

After inputting the patient’s basic data, the accuracy rate of the most likely and total diagnosis was 37 and 55%, respectively. Inputting the clinical symptoms and MRI findings yielded an accuracy of 37% for the most likely diagnosis. Inputting all paraclinical data increased accuracy to 48%; however, this improvement was not statistically significant (*p* = 0.142). Similarly, Inputs 2, 3, and 4 resulted in accuracy rates of 36% (*p* = 0.868 vs. Input 1), 47% (*p* = 0.209 vs. Input 1), and 48% (*p* = 0.142 vs. Input 1), respectively. Despite increases in diagnostic accuracy with the addition of various paraclinical data, none of these changes were statistically significant. Pairwise comparisons between outcomes after inputting the MRI description, EEG description, and CSF results also showed no significant changes (*p* = 0.178, 0.121, and 0.854, respectively).

In terms of the total diagnostic accuracy rate, the three subsequent inputs did not result in significant changes compared to the basic input (55% vs. 67%, *p* = 0.111, 55% vs. 64%, *p* = 0.251 and 55% vs. 65%, *p* = 0.193, respectively). Pairwise comparisons between outcomes after inputting the MRI description, EEG description, and CSF results also showed no significant changes (*p* = 0.743, 0.847, and 0.897, respectively).

**Figure 4:**
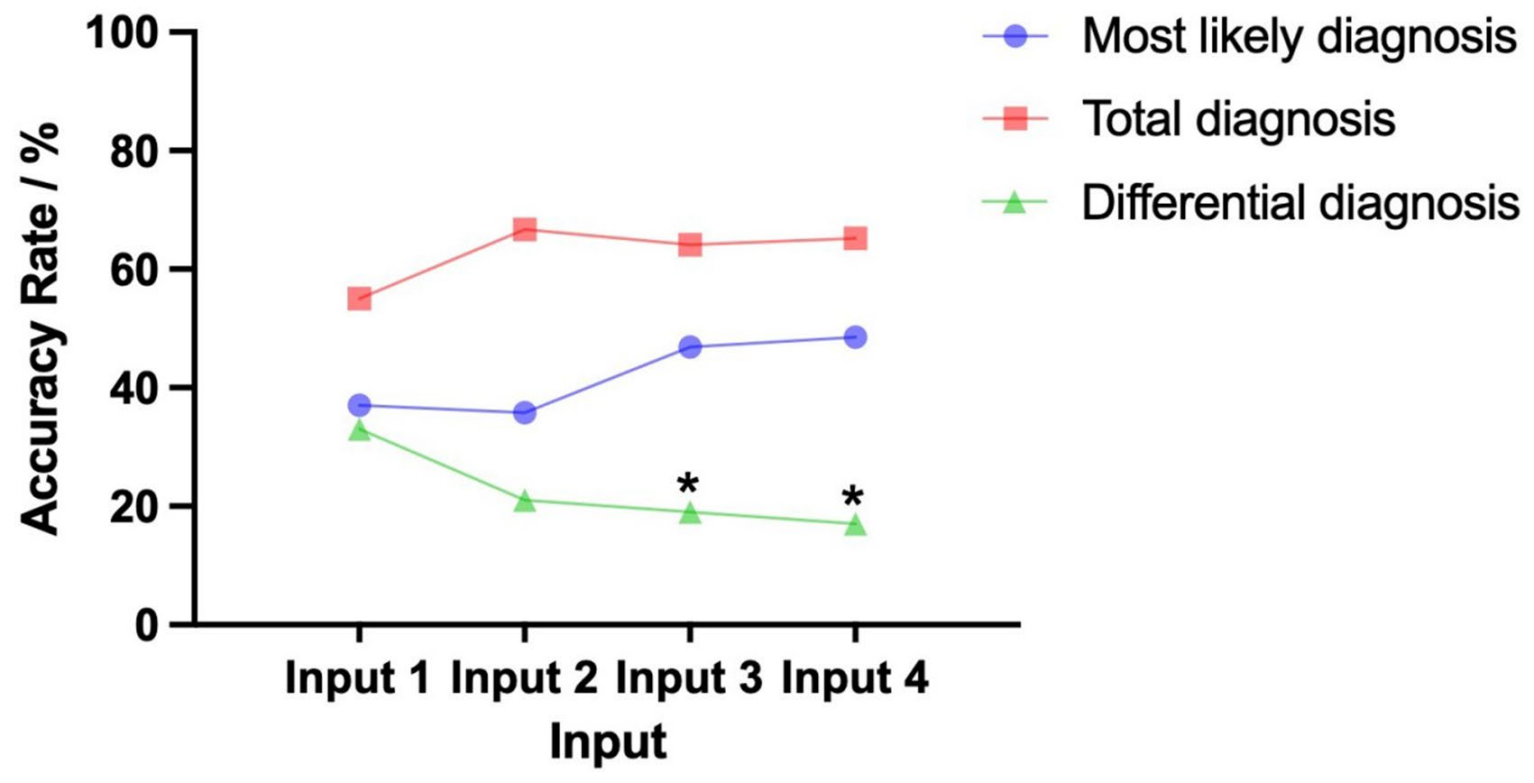
Trends of the most likely and total diagnosis accuracy rate given the basic input followed by inputting the MRI description, EEG description, and CSF results. For the 100 antibody-positive AIE patients included in the analysis, pairwise comparisons between Input 1, Input 2, Input 3, and Input 4 showed no significant differences for the total diagnosis. There were also no significant differences for the most likely diagnosis. However, for differential diagnosis, the accuracy rate of Inputs 3 and 4 was significantly lower (*p* < 0.05). We consider cases where “autoimmune encephalitis” appears in either the most likely diagnosis or differential diagnosis as a positive result. Input 1 includes basic input (sex, age, chief complaint, medical history, and vaccination or infection history). Input 2 includes basic input and MRI description. Input 3 includes basic input, MRI description, and EEG description. Input 4 includes basic input, MRI description, EEG description, and CSF results. *p < 0.05 (vs Input 1, exploratory result).

However, the accuracy rate demonstrated a contrasting trend for differential diagnosis. After further inputting the EEG description and CSF results, the accuracy rate significantly decreased compared to that for the basic input (19% vs. 33%, *p* = 0.046, and 17% vs. 33%, *p* = 0.020, respectively). Further inputting the MRI description did not significantly change the accuracy rate compared to that for the basic input (21% vs. 33%, *p* = 0.072). Pairwise comparisons between outcomes after inputting the MRI description, EEG description, and CSF nucleated cell results also showed no significant changes (*p* = 0.738, 0.507, and 0.756, respectively; Figure 4, Table 2).

**Table 2:** Diagnostic prediction accuracy of DeepSeek for antibody positive AIE patients.

Most likely diagnosis		Differential diagnosis	
Input 1^a^			
Autoimmune encephalitis	37/100 [37%]	Autoimmune encephalitis	33/100 [33%]
Viral encephalitis	23/100 [23%]	Viral encephalitis	26/100 [26%]
Metabolic/toxic encephalopathy	22/100 [22%]	Metabolic/toxic encephalopathy	25/100 [25%]
Epilepsy	22/100 [22%]		
Input 2^a^			
Autoimmune encephalitis	29/81 [36%]	Viral encephalitis	26/81 [32%]
Multiple sclerosis	18/81 [22%]	Brain tumor	19/81 [23%]
Viral encephalitis	15/81 [19%]	Autoimmune encephalitis	17/81 [21%]
Input 3^a^			
Autoimmune encephalitis	30/64 [47%]	Metabolic encephalopathy	31/64 [48%]
Viral encephalitis	14/64 [22%]	Viral encephalitis	25/64 [39%]
Epilepsy	14/64 [22%]	Epilepsy	16/64 [25%]
Input 4^a^			
Autoimmune encephalitis	32/66 [48%]	Viral encephalitis	31/66 [47%]
Viral encephalitis	16/66 [24%]	Metabolic encephalopathy	27/66 [41%]
Multiple sclerosis	6/66 [9%]	Bacterial encephalitis	14/66 [21%]

a Input 1 includes basic input (sex, age, chief complaint, medical history, and vaccination or infection history). Input 2 includes basic input and MRI description. Input 3 includes basic input, MRI description, and EEG description. Input 4 includes basic input, MRI description, EEG description, and CSF results.

### Impact of different antibody types on DeepSeek’s diagnostic accuracy

For the 100 patients included in the analysis, 17 types of AIE antibodies were detected. The most frequently detected antibodies were anti-NMDAR, anti-MOG, and anti-LGI1. Notably, the most likely and total diagnosis accuracy rates demonstrated divergence across antibodies.

The highest most likely diagnosis accuracy rate was associated with anti-MOG (88%), which was significantly higher than that of other antibody types (88% vs. 43%, *p* = 0.025). The total diagnosis accuracy rate of anti-MOG-positive patients was 88%, which showed no significant difference with that of other antibodies (88% vs. 62%, *p =* 0.244).

The highest total diagnosis accuracy rate was associated with anti-GABAbR (100%), although this was not significantly higher than that of other antibody types (100% vs. 62%, *p* = 0.084). The most likely diagnosis accuracy rate of anti-GABAbR-positive patients was (83.3%), which was not significantly different than that of other antibodies (83.3% vs. 45%, *p* = 0.077).

Patients with anti-NMDAR and anti-LGI1 had lower most likely and total diagnosis accuracy rates compared to other antibodies. The antibody anti-LGI1 had the lowest most likely and total diagnosis accuracy rate compared to other antibody types (25% vs. 54%, *p* = 0.030 and 33% vs. 72%, *p* = 0.018, respectively).

The most likely and total diagnosis accuracy rate was also lower for patients with anti-NMDAR, although the rate was not significantly different than that of other antibody types (53% vs. 47%, *p* = 0.669 and 80% vs. 61%, *p* = 0.170).

### Impact of different symptoms on DeepSeek’s diagnostic accuracy

To clarify the impact of different symptoms on DeepSeek’s diagnostic prediction, we compared the diagnosis accuracy rates between AIE patients with different clinical presentations. The clinical symptom with the highest diagnosis accuracy rate was headache, with a total diagnosis accuracy rate of 100% and a most likely diagnosis accuracy rate of 83%. The accuracy rate for AIE patients with headache was significantly higher than that for AIE patients with other related symptoms for total diagnosis (100% vs. 65%, *p* = 0.014); however, the most likely diagnosis accuracy rate was not significantly different (83% vs. 48%, *p* = 0.051).

The second highest accuracy rate was associated with the clinical symptom epilepsy, with a total diagnosis accuracy rate of 96% and a most likely diagnosis accuracy rate of 80%. The accuracy rate for AIE patients with epilepsy was significantly higher than that of other patients in terms of both total diagnosis and most likely diagnosis (96% vs. 56%, *p* = 0.001 and 80% vs. 39%, *p* = 0.001).

**Figure 5:**
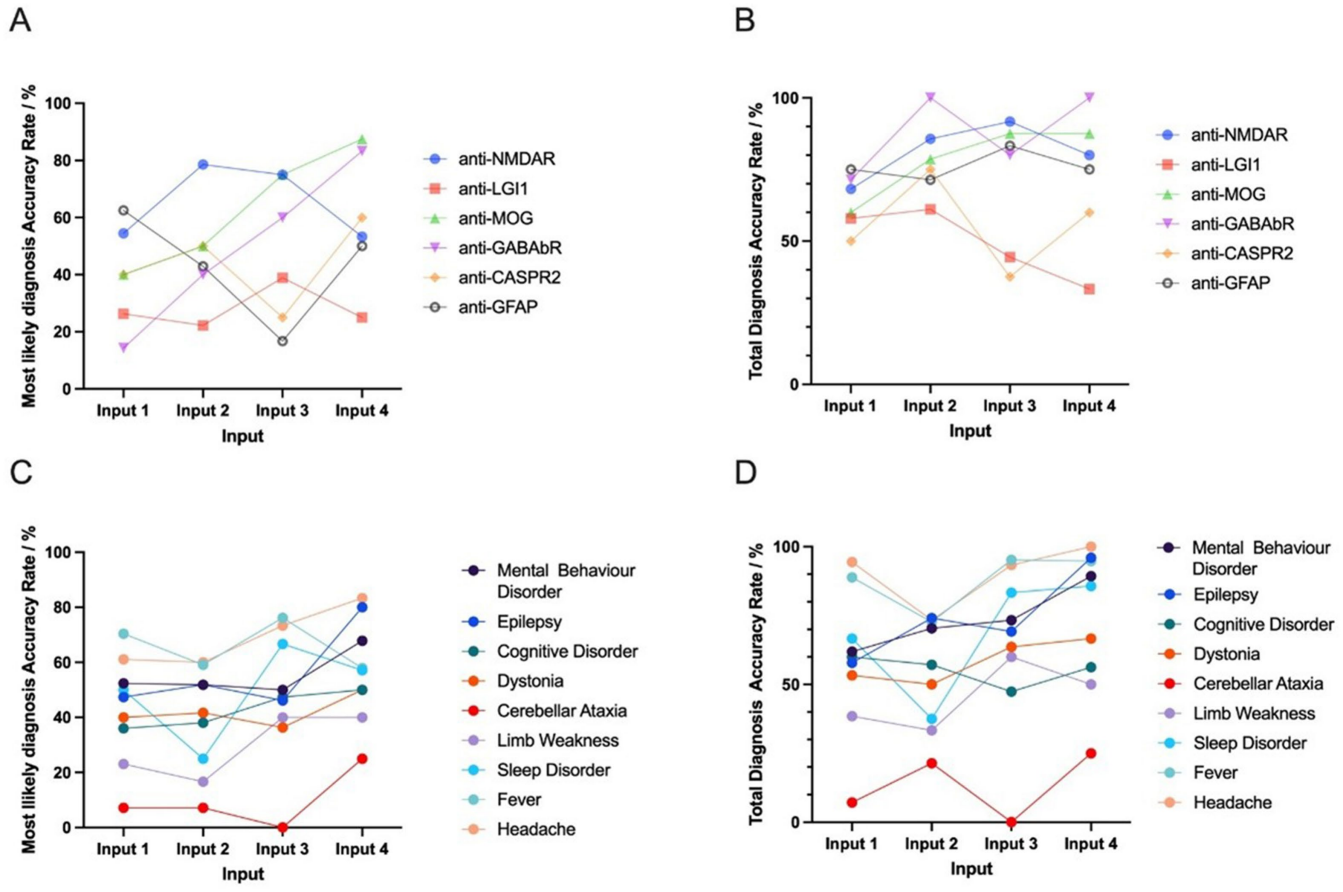
The most likely and total diagnosis accuracy rates given basic input and further input (MRI description, EEG description, and CSF results) between different antibodies and symptoms. Among the 100 antibody-positive AIE patients included in the analysis, 17 types of AIE antibodies were observed. The six highest-ranked DeepSeek-classified AIE-associated antibodies (*n* ≥ 5) that demonstrated non-negative diagnostic validity underwent comparative evaluation. **A,B** show the trend of the most likely and total diagnosis accuracy rate for different antibodies. Among the 100 AIE patients, 14 clinical manifestations were observed. The nine highest-ranked symptoms (*n* ≥ 5) demonstrating non-negative diagnostic validity underwent comparative evaluation. **C,D** indicate the trend of the most likely and total diagnosis accuracy rate for different symptoms. For statistical robustness, only subgroups with *n* ≥ 5 were included. Inter-antibody and inter-symptom diagnostic performance heterogeneity were quantified and followed by statistical validation through Input 4-based hypothesis testing (Fisher’s exact test and Chi-square test, *α* = 0.05). Input 1 includes basic input (sex, age, chief complaint, medical history, and vaccination or infection history). Input 2 includes basic input and MRI description. Input 3 includes basic input, MRI description, and EEG description. Input 4 includes basic input, MRI description, EEG description, and CSF results.

The clinical symptom with the lowest diagnosis accuracy rate was cerebellar ataxia, with a total diagnosis accuracy rate of 25% and a most likely diagnosis accuracy rate of 25%. The accuracy rate for this symptom was significantly lower than for other AIE patients in terms of total diagnosis (25% vs. 78%, *p* = 0.006). However, there was no significant difference between the most likely accuracy rate of AIE patients with and without cerebellar ataxia (25% vs. 59%, *p* = 0.128) (Figure 5).

## Discussion

This study provides the first attempt to use the AI tool DeepSeek to diagnose AIE. Due to the diverse symptoms of AIE and frequent non-specific findings in brain MRI and CSF parameters, the diagnosis of AIE is challenging. Thus, developing a method to rapidly screen and diagnose AIE is of significant clinical value. Using DeepSeek to analyze patient clinical data, the probability of AIE appearing in the most likely diagnosis was 49%. However, when differential diagnosis was considered, the total diagnosis accuracy increased to 67%. When patient data were input stepwise, including basic input, MRI image description, EEG description, and CSF results, there was no significant increase in the total diagnosis accuracy or most likely diagnosis accuracy. Notably, we found that different clinical symptoms and AIE-related antibody types may influence diagnostic accuracy. Overall, our findings suggest a higher predicted diagnostic positivity rate for AIE patients with anti-MOG and anti-GABAbR positivity. Our preliminary subgroup analysis indicated that headache or epilepsy presentation is associated with a higher likelihood of AIE diagnosis. Overall, these findings provide theoretical support for the use of AI in assisting with the diagnosis of AIE, with possible further applications in medical research and education.

### The need for applications of AI in healthcare

The term AI was coined by John McCarthy of Dartmouth College in 1956 and is now used as a general term to describe the capabilities and operations of machines (computers) that mimic or emulate human intelligence (Gore, 2020). While AI technology is being widely applied in various health-related fields, such as imaging diagnostics (Gore, 2020), heart failure diagnosis (Yasmin et al., 2021), and dermatopathology diagnosis (Wells et al., 2021), these tools have significant limitations, such as being difficult to use or requiring a certain level of programming expertise. As a result, AI has not yet achieved widespread adoption across healthcare settings.

Although there is increasing recognition of AIE by neurologists (Graus et al., 2016), its diagnosis remains challenging in primary hospitals in China. This difficulty is mainly due to reliance on antibody testing and imaging positivity rates (Abboud et al., 2021). Of the limited research on the final diagnosis rate of AIE in Chinese primary hospitals, a study involving 457 patients with suspected AIE reported a positivity rate of anti-neuronal antibody testing of 41.36% (Gu et al., 2019). Such a low antibody positivity rate severely impacts the diagnosis of AIE (Flanagan et al., 2023). The low diagnostic positivity rate in primary hospitals in China is further affected by the high cost of neuronal antibody spectrum testing, which many patients cannot afford. In economically underdeveloped regions of China, the cost of such testing often exceeds the average monthly income. The development of a rapid, cost-effective method to assist AIE diagnosis using accessible AI tools like DeepSeek is thus crucial for improving the diagnostic capabilities of primary hospitals in such areas.

### From ChatGPT to DeepSeek: new AI tools for medical applications

ChatGPT, a generative AI chatbot, is increasingly recognized as a promising tool to improve healthcare quality (Kulkarni and Singh, 2023). DeepSeek, which was launched in January 2025, has rapidly disrupted the field by offering ChatGPT-tier performance at no cost, triggering volatility in global tech markets (Kayaalp et al., 2025). A study comparing DeepSeek and ChatGPT identified several similarities between these two AI tools, including performance—with both excelling in clinical data interpretation, drafting manuscripts, and completing complex scientific tasks—and limitations, with both struggling with basic tasks and occasionally producing “hallucinations” (Yu et al., 2024). Among AI tools, DeepSeek stands out because of its transparency and accessibility. Its open-access design allows auditing, modification, and retraining for specific needs. DeepSeek is also more affordable, offering free-tier access and self-hosting options, making it more available to smaller institutions (Kayaalp et al., 2025). When AI tools are used in medical fields, there are important ethical considerations. Although AI technology can help clinicians rapidly acquire knowledge and shows potential applications in medical education and research by processing information efficiently and reducing workload, caution should be exercised when considering the extent to which AI could replace physicians in clinical diagnosis (Yu et al., 2024).

In the present study, we found that DeepSeek achieved 49% accuracy in identifying the most likely diagnosis of AIE without relying on indicative information such as the type of antineuronal antibodies or prior immunosuppressive treatments. When considering differential diagnoses, the total diagnosis accuracy reached 61%. AI tools like DeepSeek offer various applications for AIE. As demonstrated in this study, DeepSeek can predict the likelihood of AIE even when clinical information is incomplete, including for patients previously diagnosed with psychiatric disorders or primary epilepsy. However, predictions using DeepSeek should not replace formal antibody testing, tumor screening, or comprehensive clinical evaluation for AIE. Identifying the specific AIE subtype is essential for treatment planning, cancer surveillance, and prognostic assessment. As such, DeepSeek should be viewed as a tool to support—not replace—evidence-based clinical decision-making. Notably, we found that different antibody types and clinical symptoms may influence the diagnosis accuracy. This effect is likely due to the relatively distinct and well-documented symptom clusters associated with these subtypes, which may be more easily recognized by the model through its language-based reasoning. However, the underlying reasons for these differences remain unclear. We speculate that patients with different antibody types may exhibit variation in clinical presentation. As a result, some patients’ clinical characteristics may more closely resemble or differ from those in the AI tool’s training dataset, leading to higher or lower diagnosis accuracy rates for these patients. However, as the clinical characteristics of the AI tool’s training dataset change over time and make statistical analysis challenging, further research is needed to explore the diagnostic logic of DeepSeek.

### Speculations on the possible reasons for variability in DeepSeek’s predicted positivity rates for autoimmune encephalitis

Our study also identified several potential issues with DeepSeek in data processing. First, in some cases, we observed that DeepSeek tended to autonomously supplement the input data based on its pre-trained database. This sometimes led to the inclusion of biased or inferred clinical information in the diagnostic decision-making process. Although a new dialogue was initiated for each patient’s data input to minimize potential learning effects, it is not possible to entirely rule out the influence of such effects on the study’s findings.

Second, DeepSeek performed better for patients with anti-NMDAR and anti-LGI1 antibodies. This is likely because these subtypes are associated with relatively distinct and well-documented symptom clusters (e.g., psychiatric symptoms, seizures, and memory impairment), which the model may be better able to identify due to its language-based reasoning. In contrast, uncommon or less well-characterized subtypes (e.g., anti-GABAbR, anti-CASPR2) may exhibit heterogeneous or overlapping features, which have the effect of lowering diagnostic consistency.

Third, to evaluate the impact of the integrity of inputs, different input sequences and compare stepwise versus one-time data entry on diagnostic outcomes, we conducted a randomized sampling analysis. No significant differences in the positive prediction rate for AIE were observed in our study (Supplementary Tables 2,3). However, whether input sequence influences diagnostic outcomes in larger patient populations remains an open question and warrants further investigation.

Fourth, although headache is not a classical symptom of AIE, the model’s diagnostic accuracy can be attributed to contextual associations. We observed that “headache” rarely occurred in isolation in our dataset; instead, it frequently co-occurred with other neurological or psychiatric symptoms, such as fever, altered mental status, or seizures, which may have collectively led the model to infer AIE. In other words, DeepSeek might identify headache as a component of a broader constellation of early or prodromal symptoms rather than giving it a high diagnostic weight in and of itself.

Fifth, the trend that the “most likely diagnosis” positivity rate increases, but the “differential diagnosis” and “total diagnosis” rates remain stable or even decrease—is indeed a notable phenomenon. We believe this reflects several aspects of how LLM-based tools like DeepSeek operate in clinical decision support. DeepSeek, as a transformer-based LLM, functions by integrating and weighting input information based on patterns learned from its training corpus (Tordjman et al., 2025). Unlike rule-based systems, LLMs do not simply “accumulate” evidence; rather, they interpret each new piece of information in the context of the full prompt using attention mechanisms (Hernández and Amigó, 2021). This means that new information can shift the model’s diagnostic weighting in potentially non-linear or counterintuitive ways. These issues may significantly compromise the accuracy of diagnostic research for AIE and potentially mislead healthcare professionals or researchers who are less familiar with this condition.

## Limitations

This study is subject to several limitations. First, our sample excluded antibody-negative AIE patients to fully align with the current diagnostic criteria for confirmed AIE. While our data included as many types of antibody-positive patients as possible, we cannot guarantee coverage of all antibody-positive AIE cases. As a result, our study may not fully reflect DeepSeek’s diagnostic performance across all AIE subtypes. Second, as a retrospective, real-world study, clinical data were missing for some patients. As we aimed to simulate the clinical decision-making process during a physician’s initial encounter with a patient suspected of having AIE, we deliberately excluded certain clinical information that could strongly indicate an AIE diagnosis, such as the presence of anti-neuronal antibodies or the patient’s response to immunosuppressive therapy. This may have introduced bias in the calculated diagnostic accuracy. Third, differences in physicians’ understanding of AIE may lead to variation in how patient histories are recorded, even when cases have only minor clinical differences. These discrepancies in medical history documentation could result in different prediction outcomes when input into DeepSeek, potentially affecting the diagnostic positivity rate. Inherent technical characteristics of tools like DeepSeek—such as learning effects—may also lead to significant bias in the results, and such bias is difficult to eliminate. Finally, over-reliance on AI-generated output may risk misinterpretation. DeepSeek is intended for supportive use only in medical education, research, and diagnostic exploration, and is not designed to independently replace clinical judgment or serve as a standalone diagnostic instrument.

## Conclusion

This study indicates that DeepSeek, a newly developed AI chatbot, has limited positive diagnostic accuracy for predicting AIE and cannot replace clinical decision-making by physicians. Nevertheless, application of AI technology may be of potential value as an adjunctive resource for early screening in primary hospitals in China, medical education, and research advances in AIE.

## Data Availability

The original contributions presented in the study are included in the article/Supplementary material, further inquiries can be directed to the corresponding author.
