# Supplementary material for: The potential of DeepSeek for AI-aided diagnosis of antibody-positive autoimmune encephalitis: a single-center, retrospective, observational study
Source: Front Artif Intell. 2025 Oct 6;8:1638904. doi: 10.3389/frai.2025.1638904 (PMC12536004; doi:10.3389/frai.2025.1638904)
Supplement: Supplementary file 1 [file Table_1.pdf]

Supplementary Table 1. Statistical comparison of full cohort and complete-case cohort (Chi-square test)

Group	Positive	Negative	Total	χ^2	*P*	*df*
Most likely diagnosis						
Input1						
Full cohort	37	63	100			
Complete-case cohort	19	22	41	1.060	0.303	1
Total	56	85	141			
Input2						
Full cohort	29	52	81			
Complete-case cohort	18	23	41	0.754	0.868	1
Total	47	75	122			
Input3						
Full cohort	30	34	64			
Complete-case cohort	18	23	41	0.089	0.298	1
Total	48	57	105			
Input4						
Full cohort	32	34	66			
Complete-case cohort	22	19	41	0.271	0.520	1
Total	54	53	107			
Total diagnosis						
Input1						
Full cohort	55	45	100	0.148	0.701	1
Complete-case cohort	24	17	41			
Total	85	56	141			
Input2						
Full cohort	54	27	81	0.033	0.857	1
Complete-case cohort	28	13	41			
Total	89	33	122			
Input3						
Full cohort	41	23	64			
Complete-case cohort	26	15	41	0.005	0.946	1
Total	67	38	105			
Input4						
Full cohort	42	24	66			
Complete-case cohort	29	12	41	0.570	0.450	1
Total	71	36	107			
