# Supplementary material for: The potential of DeepSeek for AI-aided diagnosis of antibody-positive autoimmune encephalitis: a single-center, retrospective, observational study
Source: Front Artif Intell. 2025 Oct 6;8:1638904. doi: 10.3389/frai.2025.1638904 (PMC12536004; doi:10.3389/frai.2025.1638904)
Supplement: Supplementary file 2 [file Table_2.pdf]

Supplementary Table 2. Statistical analysis of original input sequence and an alternative order (Fisher's exact test)

Group	Positive	Negative	Total	χ^2	*P*-value	*df*
Most likely diagnosis						
Sequence1	37	63	100			
Sequence1'	3	7	10	/	0.744	/
Total	40	70	110			
Sequence1-2	29	52	81			
Sequence1-4	6	4	10	/	0.175	/
Total	35	56	91			
Sequence1-2-3	30	34	64			
Sequence1-4-3	6	4	10	/	0.510	/
Total	36	38	74			
Sequence1-2-3-4	32	34	66			
Sequence1-4-3-2	6	4	10	/	0.736	/
Total	38	38	76			
Total diagnosis						
Sequence1	55	45	100			
Sequence1'	3	7	10	/	0.187	/
Total	58	52	110			
Sequence1-2	54	27	81			
Sequence1-4	6	4	10	/	0.730	/
Total	60	31	91			
Sequence1-2-3	41	23	64			
Sequence1-4-3	8	2	10	/	0.479	/
Total	49	25	74			
Sequence1-2-3-4	42	24	66			
Sequence1-4-3-2	8	2	10	/	0.479	/
Total	50	26	76			

In this table, we randomly selected 10 patients with complete data and entered their information in the order of input1-4-3-2. We then compared the positivity rates of AIE appearing in the "most likely diagnosis" and "total diagnosis" between these 10 patients and the original dataset under different input sequences (1-2-3-4 and 1-4-3-2). Fisher's exact test was used to compare the positivity rates between the two groups.
