# Supplementary material for: The potential of DeepSeek for AI-aided diagnosis of antibody-positive autoimmune encephalitis: a single-center, retrospective, observational study
Source: Front Artif Intell. 2025 Oct 6;8:1638904. doi: 10.3389/frai.2025.1638904 (PMC12536004; doi:10.3389/frai.2025.1638904)
Supplement: Supplementary file 3 [file Table_3.docx]

**Supplementary Table 3. Statistical comparison of one-time complete input and fragmented inputs (Fisher’s exact test)**

| Group | Positive | Negative | Total | χ^2^ | *P* | *df* |
| --- | --- | --- | --- | --- | --- | --- |
| Most likely diagnosis | | | | | | |
| One-time complete | 4 | 6 | 10 | / | 0.656 | / |
| Fragmented inputs | 6 | 4 | 10 |  |  |  |
| Total | 10 | 10 | 20 |  |  |  |
| Differential diagnosis | | | | | | |
| One-time complete | 2 | 8 | 10 | / | >0.999 | / |
| Fragmented inputs | 2 | 8 | 10 |  |  |  |
| Total | 4 | 16 | 20 |  |  |  |
| Total diagnosis | | | | | | |
| One-time complete | 6 | 4 | 10 | / | 0.628 | / |
| Fragmented inputs | 8 | 2 | 10 |  |  |  |
| Total | 14 | 6 | 20 |  |  |  |

In this table, we randomly selected 10 patients diagnosed with AIE and applied two input strategies to enter their clinical data. One strategy involved a one-time complete input, where all available information—including clinical features, MRI findings, EEG descriptions, and CSF nucleated cell counts—was entered simultaneously. The other strategy involved sequential input across four steps. We compared the positivity and negativity rates of AIE appearing in the “most likely diagnosis”, “differential diagnosis”, and “total diagnosis” categories under both strategies. Fisher’s exact test was used to assess statistical differences in the distribution of these rates between the two approaches.
